# Disparities in Screening for Substance Use Among Injured Adolescents

**DOI:** 10.1001/jamanetworkopen.2024.36371

**Published:** 2024-10-04

**Authors:** Jordan M. Rook, Ryan G. Spurrier, Christopher J. Russell, Cathy E. Shin, Steven L. Lee, Catherine J. Juillard, Lorraine I. Kelley-Quon

**Affiliations:** 1 Greater Los Angeles Veterans Administration Healthcare System, Los Angeles, California; 2 Department of Surgery, David Geffen School of Medicine at University of California, Los Angeles; 3 University of California, Los Angeles National Clinician Scholars Program, Los Angeles; 4 Fielding School of Public Health at the University of California, Los Angeles; 5 Division of Pediatric Surgery, Children’s Hospital Los Angeles; 6 Department of Surgery, Keck School of Medicine at University of Southern California, Los Angeles; 7 Division of Pediatric Hospital Medicine, Stanford University School of Medicine, Palo Alto, California; 8 Division of Pediatric General and Thoracic Surgery, Seattle Children’s Hospital, Seattle, Washington; 9 Department of Population and Public Health Sciences, Keck School of Medicine at University of Southern California, Los Angeles

## Abstract

This cohort study compares rates of biochemical substance use screening among injured adolescents by sociodemographic characteristics including sex, race, ethnicity, and insurance status.

## Introduction

Injury and substance use are leading causes of adolescent death.^1^ Substance use screening can reduce future substance use and risk for reinjury.^2^ The American College of Surgeons (ACS) requires alcohol screening for 80% or more of trauma patients. Drug screening is recommend but not required.^2,3^ Among adult trauma patients, studies demonstrate disparate screening rates by race and ethnicity.^4^ Among adolescents, it is unknown if screening is deployed equitably. This study examined a national sample of adolescents who were injured to identify sociodemographic disparities in biochemical substance use screening.

## Methods

This retrospective cohort study used the 2017 to 2021 ACS Trauma Quality Programs (TQP) dataset. This study was deemed exempt due to the use of deidentified data by the University of California Los Angeles Institutional Review Board and followed Strengthening the Reporting of Observational Studies in Epidemiology (STROBE) reporting guideline. We identified adolescent trauma patients aged 12 to 17 years presenting to 121 ACS-verified pediatric trauma centers. We excluded patients with missing data. We assessed receipt of biochemical alcohol and drug screening as binary outcomes. We used standardized differences to evaluate baseline covariate balance, and logistic regression mixed effects modeling to evaluate associations between sociodemographic variables (ie, race, ethnicity, biological sex, insurance) and screening (eMethods in Supplement 1). Race and ethnicity were categorized per the National Trauma Data Standard and reflected self-report or identification by a family member. Race categories included American Indian, Asian, Black, Pacific Islander, White, and other. Ethnicity categories included Hispanic and non-Hispanic. Models included fixed effects for age, emergency department disposition, injury intent and mechanism, Glasgow Coma Scale, Injury Severity Score, hospital teaching status, ACS-verification, and year. Trauma center random effects nested patients within hospitals to account for institutional screening practices. Data were analyzed from February 2023 to July 2024 using Stata BE version 17.0 (StataCorp). Tests were 2-sided, and statistical significance was set at *P* < .05.

## Results

Of 85 362 adolescents, 56 901 (66.7%) were White individuals, 69 850 (81.8%) were non-Hispanic, 61 490 (72.0%) were male, and 43 818 (51.3%) were privately insured. (Table 1). Overall, 20 956 adolescents (24.5%) underwent biochemical alcohol screening. Adolescents who were Black (adjusted odds ratio [AOR], 1.08; 95% CI, 1.01-1.15) or American Indian (AOR, 2.17; 95% CI, 1.76-2.69) were more likely screened than White adolescents, and Hispanic adolescents were more likely to be screened than non-Hispanic adolescents (AOR, 1.20; 95% CI, 1.11-1.29). Medicaid insured (AOR, 1.15; 95% CI, 1.09-1.21) and uninsured (AOR, 1.13; 95% CI, 1.03-1.24) adolescents were more likely screened than those privately insured. Female adolescents (AOR, 1.32; 95% CI, 1.26-1.38) were more likely screened than males.

**Table 1.** Sociodemographic Characteristics of Adolescents by Receipt of Alcohol Screening and Drug Screening

Characteristic	Total (n = 85 362)	Alcohol screening			Drug screening		
		No (n = 64 406)	Yes (n = 20 956)	STD^a^	No (n = 66 729)	Yes (n = 18 633)	STD
Race^b^							
American Indian	868 (1.0)	562 (0.9)	306 (1.5)	0.09	559 (0.8)	309 (1.7)	0.10
Asian	1477 (1.7)	1164 (1.8)	313 (1.5)		1171 (1.8)	306 (1.7)	
Black	16 971 (19.9)	12 724 (19.8)	4247 (20.3)		13 329 (20.0)	3642 (19.6)	
Pacific Islander	202 (0.2)	161 (0.3)	41 (0.2)		164 (0.3)	38 (0.2)	
White	56 901 (66.7)	43 346 (67.3)	13 555 (64.7)		44 785 (67.1)	12 116 (65.0)	
Other^c^	8943 (10.5)	6449 (10.0)	2494 (11.9)		6721 (10.1)	2222 (11.9)	
Ethnicity							
Hispanic	15 512 (18.2)	11 000 (17.1)	4512 (21.5)	0.12	11 124 (16.7)	4388 (23.6)	0.17
Non-Hispanic	69 850 (81.8)	53 406 (82.9)	16 444 (78.5)		55 605 (83.3)	14 245 (76.5)	
Sex							
Male	61 490 (72.0)	47 055 (73.1)	14 435 (68.9)	0.09	48 685 (73.0)	12 805 (68.7)	0.09
Female	23 872 (28.0)	17 351 (26.9)	6521 (31.1)		18 044 (27.0)	5828 (31.3)	
Insurance							
Private	43 818 (51.3)	33 642 (52.2)	10 176 (48.6)	0.11	35 186 (52.7)	8632 (46.3)	0.13
Medicaid	32 549 (38.1)	23 908 (37.1)	8641 (41.2)		24 612 (36.9)	7937 (42.6)	
Uninsured	5139 (6.0)	3863 (6.0)	1276 (6.1)		3983 (6.0)	1156 (6.2)	
Medicare	2896 (3.4)	2333 (3.6)	563 (2.7)		2233 (3.4)	663 (3.6)	
Other	960 (1.1)	660 (1.0)	300 (1.4)		715 (1.1)	245 (1.3)	
Age, median (IQR)	14 (13-16)	14 (13-16)	15 (14-17)	0.57	14 (13-16)	15 (14-16)	0.49
Mechanism							
MVC	40 445 (47.4)	28 769 (44.7)	11 676 (55.7)	0.57	30 251 (45.3)	10 194 (54.7)	0.45
Fall	20 943 (24.5)	18 474 (28.7)	2469 (11.8)		18 258 (27.4)	2685 (14.4)	
Bicycle	5641 (6.6)	4428 (6.9)	1213 (5.8)		4607 (6.9)	1034 (5.6)	
Firearm	5434 (6.4)	2974 (4.6)	2460 (11.7)		3435 (5.2)	1999 (10.7)	
Auto vs pedestrian	3482 (4.1)	2248 (3.5)	1234 (5.9)		2440 (3.7)	1042 (5.6)	
Stab	3160 (3.7)	2233 (3.5)	927 (4.4)		2321 (3.5)	839 (4.5)	
Motorcycle	1148 (1.3)	695 (1.1)	453 (2.2)		790 (1.2)	358 (1.9)	
Bite or sting	1069 (1.3)	993 (1.5)	76 (0.4)		989 (1.5)	80 (0.4)	
Other	4040 (4.7)	3592 (5.6)	448 (2.1)		3638 (5.5)	402 (2.2)	
GCS, median (IQR)	15 (15-15)	15 (15-15)	15 (15-15)	0.33	15 (15-15)	15 (15-15)	0.33
ISS, median (IQR)	5 (4-10)	5 (4-9)	9 (4-14)	0.41	5 (4-9)	9 (4-14)	0.37
ACS pediatric verification							
Level 1	59 038 (69.2)	46 532 (72.3)	12 506 (59.7)	0.27	47 262 (70.8)	11 776 (63.2)	0.16
Level 2	26 324 (30.8)	17 874 (27.8)	8450 (40.3)		19 467 (29.2)	6857 (36.8)	

Abbreviations: ACS, American College of Surgeons; GCS, Glasgow Coma Scale; ISS, Injury Severity Score; MVC, motor vehicle collision; STD, standardized difference.

^a^ Standardized differences greater than 0.10 indicate covariate imbalance between groups (eMethods in Supplement 1).

^b^ Race and ethnicity were categorized per the National Trauma Data Standard and reflected self-report or identification by a family member.

^c^ The other race category includes individuals who identified as other race. Of the 8943 patients who identified as other race, 6277 (70.2) identified as Hispanic ethnicity.

Overall, 18 633 (21.8%) underwent biochemical drug screening. Adolescents who were Black (AOR, 1.13; 95% CI, 1.07-1.20) or American Indian (AOR, 1.75; 95% CI, 1.45-2.11) were more likely screened than White adolescents, and Hispanic adolescents were more likely to be screened than non-Hispanic adolescents (AOR, 1.20; 95% CI, 1.11-1.29). Medicaid-insured (AOR, 1.28; 95% CI, 1.22-1.34) and uninsured (AOR, 1.18; 95% CI, 1.08-1.30) adolescents were more likely screened than those privately insured. Female adolescents (AOR, 1.28; 95% CI, 1.23-1.34) were more likely screened than males (Table 2).

**Table 2.** Adjusted Associations Between Sociodemographic Characteristics of Adolescents and Receipt of Alcohol Screening and Drug Screening^a,b^

Characteristic	Alcohol screening		Drug screening	
	Adjusted OR (95% CI)	*P* value	Adjusted OR (95% CI)	*P* value
Race				
American Indian	2.17 (1.76-2.69)	<.001	1.75 (1.45-2.11)	<.001
Asian	0.84 (0.71-0.99)	.04	0.85 (0.72-1.00)	.04
Black	1.08 (1.01-1.15)	.02	1.13 (1.07-1.20)	<.001
Pacific Islander	0.63 (0.39-1.00)	.05	0.74 (0.48-1.16)	.19
White	1 [Reference]	NA	1 [Reference]	NA
Other^c^	1.02 (0.94-1.11)	.63	1.04 (0.96-1.12)	.38
Ethnicity				
Hispanic	1.20 (1.11-1.29)	<.001	1.12 (1.05-1.20)	.001
Non-Hispanic	1 [Reference]	NA	1 [Reference]	NA
Sex				
Female	1.32 (1.26-1.38)	<.001	1.28 (1.23-1.34)	<.001
Male	1 [Reference]	NA	1 [Reference]	NA
Insurance				
Medicaid	1.15 (1.09-1.21)	<.001	1.28 (1.22-1.34)	<.001
Uninsured	1.13 (1.03-1.24)	.01	1.18 (1.08-1.30)	<.001
Medicare	0.90 (0.78-1.03)	.14	1.07 (0.93-1.22)	.35
Private	1 [Reference]	NA	1 [Reference]	NA
Other	0.93 (0.76-1.14)	.48	1.01 (0.83-1.24)	.89

Abbreviations: NA, not applicable; OR, odds ratio.

^a^ Results of a mixed effects model comprised of fixed effects (race, ethnicity, sex, health insurance, age, emergency department disposition, injury intent, mechanism, Glasgow Coma Scale, Injury Severity Score, hospital teaching status, pediatric verification level, adult verification level, and year) and random effects (trauma center). Includes all 85 362 patients.

^b^ There were no statistically significant interactions between race and ethnicity and race and biological sex.

^c^ Other race includes individuals who self-identified as other.

## Discussion

In this national study of pediatric trauma centers, rates of biochemical alcohol and drug screening were disproportionately higher among adolescents who were American Indian, Black, Hispanic, female, Medicaid-insured, or uninsured. These differences may reflect clinician biases as inequities persisted despite adjusting for clinical characteristics and after nesting patients within trauma centers to account for institutional screening practices.

Our findings indicate potential disparities in biochemical substance use screening at pediatric trauma centers. With drug overdose and poisoning the third leading cause of childhood death, high-quality screening is critical for both injury prevention and public health. Future research should explore standardized screening protocols with defined criteria for biochemical- vs interview-based screening. This study has limitations. First, the TQP dataset does not report whether a positive test is followed by intervention or treatment.^2^ Thus, it remains unclear if the benefits of screening outweigh potential harms including stigmatization by clinicians and legal consequences.^5,6^ While the ACS requires an intervention for 80% of patients who screen positive for alcohol, they recommend but do not require it following a positive drug screen.^2,3^ Second, because the TQP dataset does not monitor interview-based substance use screening, we assessed only biochemical screening, not overall rates of screening. Third, it is unclear whether disparities differ by hospital case-mix (ie, high- vs low-proportion of patients from racially and ethnically minoritized backgrounds). Future iterations of the TQP dataset should report receipt of interview-based screening and subsequent treatments. The inclusion of these datapoints would enable the expansion of high-quality screening but with equity across adolescents.

## Notes

Supplement 1.
eMethods.

eReferences.

Supplement 2.
Data Sharing Statement
