# Supplementary material for: Disparities in Screening for Substance Use Among Injured Adolescents
Source: JAMA Netw Open. 2024 Oct 4;7(10):e2436371. doi: 10.1001/jamanetworkopen.2024.36371 (PMC11452809; doi:10.1001/jamanetworkopen.2024.36371)
Supplement: Supplement 1. — eMethods. eReferences. [file jamanetwopen-e2436371-s001.pdf]

Supplemental Online Content

Rook JM, Spurrier RG, Russell CJ, et al. Disparities in screening for substance use among injured adolescents. *JAMA Netw Open*. 2024;7(10):e2436371.
doi:10.1001/jamanetworkopen.2024.36371

eMethods.

eReferences

This supplemental material has been provided by the authors to give readers additional information about their work.

© 2024 Rook JM et al. *JAMA Network Open.*

eMethods

This retrospective cohort study used the 2017-2021 ACS Trauma Quality Programs (TQP) dataset. This national trauma registry includes patients cared for at most ACS-verified and many non-ACS verified pediatric trauma centers. For ACS-verified pediatric trauma centers, data collection is standardized according to the National Trauma Data Standard, performed by trained trauma registrars, and periodically audited by the ACS to ensure accuracy.[1-3] This study was deemed exempt by the UCLA Institutional Review Board and followed the STROBE reporting guidelines.

We identified adolescent trauma patients (12-17 years) presenting to 121 pediatric ACS-verified trauma centers. Patients with any missing data were excluded (n=19,338; 18.5%). We assessed receipt of biochemical alcohol and drug screening as separate binary outcomes. Receipt of biochemical alcohol screening and receipt of biochemical drug screening are both mandatory fields in the TQP dataset. While drug screening is not mandatory for ACS verification, trauma centers are required to report 1) whether a patient received a biochemical drug screen and 2) the results of that drug screen. There were no patients with missing outcome data for urine drug screening. There were 684 patients (0.6%) who were missing alcohol screening data. We used standardized differences to compare the distribution of baseline characteristics by each outcome. A standardized difference greater than 0.1 indicates covariate imbalance between groups.[4]

We evaluated associations between sociodemographic variables (race, ethnicity, biological sex, insurance) and screening with logistic regression mixed effects modeling. Covariates were selected using a conceptual model and based on prior research.[5-6] Models were adjusted for the fixed effects of age, emergency department disposition, injury intent and mechanism, Glasgow Coma Scale (GCS), Injury Severity Score (ISS), hospital teaching status, ACS verification level, and year. Race and ethnicity were categorized per the National Trauma Data Standard and reflected self-report or identification by a family member.[3] ISS and GCS were assessed as categorical variables given potential non-linear relationships with the outcomes. We used trauma center random effects with random intercepts and slopes to nest patients within trauma centers and account for differences in institutional screening practices. We did not use any hospital-level variables other than trauma center ID as a clustering variable. The random intercept and slope model by trauma center ID had significantly better model fit on likelihood ratio test (p<0.001) than a model with random intercepts only. Furthermore, given the intersectionality of race and ethnicity and race and gender, we assessed interaction terms between these covariates. No interaction reached statistical significance, and these interactions did not improve model fit by likelihood ratio tests (p=0.84) nor Akaike Information Criteria. Thus, these interaction terms were not included as fixed effects. To test the robustness of our findings to various model specifications we conducted a sensitivity analysis with only patients who were admitted from the ED (n=68,345; 80.1%). The results of this sensitivity analysis were largely unchanged from data generated using the entire study cohort.

© 2024 Rook JM et al. *JAMA Network Open.*

eReferences

1. Resources for Optimal Care of the Injured Patient. ACS. Accessed October 28, 2023. https://www.facs.org/quality-programs/trauma/quality/verification-review-and-consultation-program/standards/

2. Shafi S, Nathens AB, Cryer GH, et al. The Trauma Quality Improvement Program of the American College of Surgeons Committee on Trauma. *Journal of the American College of Surgeons*. 2009;209(4):521. doi:10.1016/j.jamcollsurg.2009.07.001

3. ACS Committee on Trauma. *National Trauma Data Standard- Data Dictionary- 2021 Admissions*. American College of Surgeons; 2022

4. Austin PC. Using the Standardized Difference to Compare the Prevalence of a Binary Variable Between Two Groups in Observational Research. *Communications in Statistics - Simulation and Computation*. 2009;38(6):1228-1234. doi:10.1080/03610910902859574

5. Silver CM, Visenio MR, Thomas AC, et al. Hospital variability in adoption of alcohol and drug screening in adult trauma patients. *J Trauma Acute Care Surg*. 2023;94(5):684. doi:10.1097/TA.0000000000003928

6. Alser O, Perez NP, Gebran A, et al. Underuse and Variability in Substance Use Screening Among Adult Trauma Patients in the U.S. – A Nationwide Retrospective Cohort Study. *Annals of Surgery*. Published online February 17, 2022. doi:10.1097/SLA.0000000000005331

© 2024 Rook JM et al. *JAMA Network Open.*
