# Supplementary material for: Disparities in Screening for Substance Use Among Injured Adolescents
Source: JAMA Netw Open. 2024 Oct 4;7(10):e2436371. doi: 10.1001/jamanetworkopen.2024.36371 (PMC11452809; doi:10.1001/jamanetworkopen.2024.36371)
Supplement: Supplement 2. — Data Sharing Statement [file jamanetwopen-e2436371-s002.pdf]

Data Sharing Statement

Data
Data available: No

Additional Information
Explanation for why data not available: The data used in this research is property of the American College of Surgeons which prevents sharing. Data can be obtained by request to the American College of Surgeons.
